# The Omicron (B.1.1.529) SARS-CoV-2 variant of concern also affects companion animals

**DOI:** 10.3389/fvets.2022.940710

**Published:** 2022-08-12

**Authors:** Lidia Sánchez-Morales, José M. Sánchez-Vizcaíno, Marta Pérez-Sancho, Lucas Domínguez, Sandra Barroso-Arévalo

**Affiliations:** ^1^VISAVET Health Surveillance Centre, Complutense University of Madrid, Madrid, Spain; ^2^Department of Animal Health, Faculty of Veterinary, Complutense University of Madrid, Madrid, Spain

**Keywords:** Omicron variant, B.1.1.529, SARS-CoV-2, companion animals, RT-qPCR

## Abstract

The emergence of the Omicron variant (B.1. 1.529) has brought with it an increase in the incidence of SARS-CoV-2 disease. However, there is hardly any data on its incidence in companion animals. We have detected the presence of this new variant in domestic animals (dogs and cats) living with infected owners in Spain. None of the RT-qPCR positive animals (10.13%) presented any clinical signs and the viral loads detected were low. In addition, the shedding of viral RNA lasted a short period of time in the positive animals. Infection with this variant of concern (VOC) was confirmed by RT-qPCR and sequencing. These outcomes suggest a lower virulence of this variant in infected cats and dogs. They also demonstrate the transmission from infected humans to domestic animals and highlight the importance of active surveillance as well as genomic research to detect the presence of VOCs or mutations associated with animal hosts.

## Introduction

The pandemic associated with the Corona Virus Disease 2019 (COVID-19), produced by the SARS-CoV-2 virus, and has been active for almost 2 years now. To the date, more than 400 million cases have been confirmed in the world with more than 6 million deaths according to the last World Health Organization (WHO) report (1). The causative agent of the pandemic, the SARS-CoV-2 virus is an RNA virus whose organization is shared with other Beta coronaviruses. The genome of this virus consists of 13 opening reading frames (ORFs) and 15 non-structural proteins (NSP). The ORFs, from 5' to 3', codify for replicase (ORF1a/ORF1b), spike (S) protein, envelope (E) protein, membrane (M) protein, and nucleocapsid (N) protein (2–4).

Due to the increased ability of RNA viruses to accumulate mutations, it has been undergoing changes such as the D614G mutation which has been associated with enhanced infectivity (5) and neuroinvasive activity, entering the CNS (Central Nervous System) *via* the olfactory nerve (6–8). The majority of the mutations are located in the spike protein, which may have influenced the virus transmission rate, the disease severity, or abrogate the immunity produced by the vaccines, among other factors (9). The emergence of these mutations has triggered virus evolution and consequently to the appearance of new variants. According to the pathogenic potential and the virulence of the different isolates, the WHO have classified them into variants of concern (VOCs), variants of interest (VOIs), and variants under monitoring (VUMs) (10). Until December 2021, four VOCs had been reported: Alpha (B.1.1.7), Beta (B.1.351), Gamma (P.1), and Delta (B.1.617.2) (11).

On November 26^th^ 2021, a new variant was determined by the WHO as the 5^th^ VOC, named Omicron (B.1.1.529). The first sample identified as this variant was taken in the South Africa's Gauteng province on the 9^th^ of November 2021, while the first sequenced case was from a sample collected in Botswana on the 11^th^ of November 2021 (10). By the 15^th^ December 2021, this variant had already emerged in 77 countries, being the majority of the cases in United States, South Africa and United Kingdom (12).

In Spain, according to data published by the Ministry of Health, the cumulative incidence of SARS-CoV-2 rose from 77 cases per 100,000 inhabitants on 15^th^ November 2021 to 465 on 15^th^ December 2021, showing a significant increase. It continued growing until reaching 3,418 on January 20^th^, 2022. This growth in cases coincided with the introduction of the Omicron variant in Spain, around mid-December 2021. As could be expected, sequencing since that time demonstrated the increasing dominance of this VOC in the country. In December 13^th^ 2021, a 5.38% of the samples sequenced in the country belonged to the Omicron variant while in March 7^th^ 2022 it raised to 99.13% (13, 14).

Until now, the Omicron variant is the VOC with the largest number of mutations detected, with 34 of them accumulated in the spike protein. Several of these mutations in the spike protein have been related to increased viral antibody neutralization evasion capacity or higher affinity between the spike/angiotensin-converting enzyme 2 (ACE2) receptor binding (9), facilitating the virus entry into the cell. Thus, this constellation of mutations appears to have influenced virus transmissibility, severity, and immune evasion (10, 15, 16). These changes have led to greater contagiousness than the previous variants as well as different clinical signs, which consist of slight fever, myalgia, fatigue, and shortness of breath (17). However, the most dangerous characteristic of this variant is its high rate of immune escape even in previously immunized by natural infection and vaccinated people (18). Because of these characteristics, the Omicron variant has gained great concern in public health worldwide.

Within the Omicron variant, five lineages or subvariants are distinguished so far: BA.1, BA.2, BA.3, BA.4 and BA.5. A total of 18 BA.1 and 27 BA.2 central mutations (frequency >99%) were identified, of which 15 are specific of the variant Omicron. Indeed, BA.2 lineage has 32 mutations shared with BA.1, but 28 mutations distinct from BA.1, and BA.3 spike protein is a combination of BA.1 and BA.2 with no new mutations. BA.2 has been observed to reinfect patients previously infected with BA.1, being more prevalent in Denmark (19–21). The emergence of the BA.1 lineage in South Africa is scheduled for mid-November 2021, while already in the week of 5^th^ of December, the proportion of the BA.2 lineage rose to 84% (22). Recently, two new Omicron lineages have been identified in South Africa, the BA.4 and BA.5, which are estimated to have originated in December 2021 (BA.4) and January 2022 (BA.5) and became the dominant variants in the country by May 2022 (23). In Spain, BA.4 and BA.5 represented more than 10% of the samples analyzed in 10 Autonomous Communities at the end of May-beginning June. These two new subvariants have some additional mutations in the spike as compared to the BA.2 lineage: 69–70del, L452R, F486V and wild type amino acid at position Q493 (24).

Shortly after the SARS-CoV-2 virus entered our lives, field studies on the incidence of this virus in animals, as well as experimental studies, began to be carried out to learn about their role in this new disease (25). In the case of animals, several studies have found that some species such as *Canis lupus familiaris, Felis catus, Nevison vison, Manis javanica, Mesocricetus auratus, Mus musculus* and *Odocoileus virginianus* are susceptible to the Omicron variant, as revealed by sequencing results from natural or experimental infection [Global Initiative on Sharing All Influenza Data (GISAID)]. However, experimental studies on hamsters have evidenced a lower pathogenicity of this variant in comparison with Delta and B.1.1 variants in this species, based on different variables such as body weight and respiratory function (26). The conclusions obtained by these studies were that, unlike other VOCs, Omicron is not able to efficiently replicate in the lower respiratory tract of Syrian hamsters, which results in the detection of lower viral loads and fewer pathology findings in the lungs of the experimentally infected animals comparing with infection with other isolates (27). In addition, inoculated mice with Omicron had lower levels of pro-inflammatory cytokines and chemokines, on occasions similar to non-infected mice, than those inoculated with the B.1.351 (Beta) variant (28). In line with these studies, another experimental investigation was recently carried out in cats. In this study, the reduced replication of Omicron in cat tissues, the limited lung inflammation and viral shedding in this specie in comparison to other variants such as B.1 D614G and Delta variant was demonstrated (29).

Despite these results suggesting a lower virulence of this variant in infected animals, very different results were observed in an experimental study in wild carnivores (mink, *Neovison vison*) which are known to be very susceptible to SARS-CoV-2 virus infection. In this study, minks were infected with the Omicron variant and consequently, they became ill, presented clinical symptoms, positive PCR results, as well as macroscopic and microscopic lesions post mortem (30). All these previous findings lead us to wonder what will be the relevance of the infection with the Omicron variant in species in close contact with humans such as dogs and cats in comparison with the previously described VOCs (31–33). Is transmissibility to susceptible pets higher with this variant, as is occurring in the case of humans? What are the clinical repercussions of the infection in cats and dogs? To elucidate the implications of infection with the Omicron variant in pets, we have carried out an active sampling of cats and dogs in close contact with SARS-CoV-2 infected people with clinical signs compatible with this variant and/or confirmed by RT-qPCR or sequencing. In this study, we have observed a low prevalence of infection in the animals, as well as low viral loads in the positive cases, despite the samplings were carried out at the optimum time point to detect human-to-pet transmission.

## Materials and methods

### Animal and owner sample collection

Samples from domestic animals including cats (*n* = 28), dogs (*n* = 50), and rabbit (*n* = 1) were taken between the 15^th^ of December 2021 to 24^th^ of March 2022. A total of 69 animals (21 cats and 47 dogs) were from Madrid, 6 animals (3 cats and 3 dogs) from Galicia, and 4 cats from the Basque Country. All these animals were sampled during the quarantine period of their owners and, therefore, had been in contact with positive people for SARS-CoV-2. The samples were taken using protocols approved by the Complutense University of Madrid's Ethics Committee for Animal Experiments (Project License 14/2020). Owners were informed about the purpose of the study as well as the data protection policy. When possible, samples were taken on 4–5 consecutive days since the beginning of the disease in the owner to gather more information about the potential animal infection. The samples consisted of oral/nasal and rectal swabs collected in DeltaSwab® Virus containing 3 ml of viral transport media (MTV) (Deltalab S.L., Cataluña, Spain) and sera if possible that were collected in tubes without anticoagulant. All the samples were refrigerated and taken to the Health Surveillance Center (VISAVET) at the Complutense University of Madrid and stored at −80°C until analysis. In addition, an epidemiological survey of the owners was carried out in order to know the potential symptoms they were presenting to confirm Omicron variant associated signs, as well as a nasal swab sample collection in some cases to confirm the SARS-CoV-2 variant involved in the infection by RT-qPCR and sequencing. They were also asked about their pets' habits to know the amount of contact with them during the illness as well as the presence or absence of compatible symptoms in their pets.

### Detection of SARS-CoV-2 infection by reverse transcription-quantitative PCR and Omicron-specific RT-qPCR and virus isolation

Total RNA was extracted using the column-based High Pure Viral Nucleic Acid Kit (Roche, Basel, Switzerland), according to the manufacturer's instructions. Total RNA was suspended in RNase/DNase-free water and stored at−80°C. The detection of the RNA of SARS-CoV-2 was carried out using a diagnostic RT-qPCR, hereafter “Diagnosis PCR”, based on the detection of the envelope protein (E)-encoding gene (Sarbeco) and two targets (IP2 and IP4) of the RNA-dependent RNA polymerase gene (RdRp) in an RT-qPCR protocol established by the World Health Organization according to the guidelines that can be found at https://www.who.int/emergencies/diseases/novel-coronavirus-2019/technical-guidance/laboratory-guidance (34).

Absolute quantification was carried out by generating a standard curve. For this purpose, a standard stock was provided by the Pasteur Institute corresponding to a load of 10^9^ copies/μl. Subsequently, serial dilutions were performed and tested in triplicate in a RT-qPCR assay to generate a standard curve with a calculated R^2^ value of 0.9983 for Sarbeco, 0.9994 for IP2 and 0.9928 for IP4.

A specific RT-qPCR was used for the identification of the SARS-CoV-2 Omicron variant, hereafter “Omicron PCR,” targeting both the envelope protein (E) - encoding gene as well as an Omicron-specific spike insertion-deletion mutation (indel_211-214) found in the B.1.1.529/BA.1 lineage and BA.1.1 sublineage, so in the case of the BA.2 and BA.3 Omicron lineages would only be detected by the gen E target. The kit used was the SuperScript III Platinum One-Step qRT-PCR kit (Invitrogen) according to the protocol described in (35).

Positive samples for RT-qPCR were subjected to attempts of viral isolation using the previously described methods in (36).

### Whole-genome sequencing and phylogenetic analysis

Whole-genome sequences were obtained from the two positive oropharyngeal swabs samples with the higher viral loads based on copies/μl (2.82 x 10^3^and 1.31 x 10^4^) by both “Diagnosis” and “Omicron” RT-qPCRs, following the protocol described by (37). Sequence analysis was performed using the Sequencing Analysis software v.5.3.1(Applied Biosystems), while SeqScape v.2.5 software (Applied Biosystems) was used for sequence assembly using the SARS-CoV-2 isolate Wuhan-Hu-1, complete genome (GenBank accession number: NC_045512) as a reference genome.

Phylogenetic analysis was performed using MEGA X software (38). Four sequences were obtained from this study (Dog_ 8, Cat_19, Owner_1, and Owner_2), which correspond with one dog, one cat, the dog's owner, and the owner of Cat_26, 27, and 28. Unfortunately, no positive sample for sequencing was available from the owner of Cat_19. In the case of cats 26, 27, and 28, sequencing was not possible because of the low RNA loads of the positive samples (Table 1).

**Table 1:** Virus detection in canine and feline patients.

**Animal, date of collection**	**Sample type**	**DPI owner**	**Diagnosis RT-qPCR copies/μl**	**Omicron RT-qPCR copies/μl**	**Sequence**	**Viral isolation**
Cat_2, 20^th^ December, 2021	Rectal swab	2 DPI	9.34 x 10^2^	1.76 x 10^2^	NA	Negative
Dog_8, 1^st^ January, 2022	Oropharyngeal swab	3 DPI	2.82 x 10^3^	1.68 x 10^2^	B.1.1.529	Negative
Cat_7, 16^th^ January, 2022	Oropharyngeal swab	2 DPI	2.06 x 10^2^	83.4	NA	Negative
Cat_19, 22^th^ January, 2022	Oropharyngeal swab	3 DPI	1.31 x 10^4^	2.29 x 10^3^	B.1.1.529	Negative
Cat_13, 27^th^ January, 2022	Oropharyngeal swab	4 DPI	3.63 x 10^2^	1.05 x 10^2^	NA	Negative
Cat_26, 18^th^ March, 2022	Oropharyngeal swab	2 DPI	7.74 x 10^2^	30.01	NA	Negative
Cat_27, 18^th^ March, 2022	Oropharyngeal swab	3 DPI	3.86 x 10^2^	43,82	NA	Negative
Cat_28, 19^th^ March, 2022	Oropharyngeal swab	4 DPI	5.64 x 10^2^	92,85	NA	Negative

*Data related to patient species, date of sampling and relationship to timeframe of the owners' first positive test, and sample type, is shown for animals testing positive by two different RT-qPCR assays as described in methods*.

A total of 31 additional representative sequences were used for the analysis, including sequences from cats and dogs, the reference genome from Wuhan, as well as variants of concern such as the B.1.1.7 variant from the United Kingdom, variant B.1.35 from South Africa, variant B.1.617.2 from India, variant B.1.1.248 from Brazil and lineages BA.1 and BA.2 of the B.1.1.529 Omicron variant.

The final alignment involved 35 whole-genome sequences with an average amino acid p-distance (1-amino acid identity) lower than 0.001, which is considered adequate since it is within the acceptance threshold of <0.8 (38). This alignment was used to build the phylogenetic tree using the maximum likelihood method and bootstrap testing of 2,000 replicates. The best model was the Tamura-Nei Model, so it was the one used to create the phylogenetic tree.

### Analysis of the mutations presented in the sequences

An analysis of the mutations present in the obtained sequences was carried out by comparing them with the reference strain of the original variant (Wuhan). This analysis was done in GISAID's CoVsurver mutations App.

### Screening enzyme-linked immunosorbent assay based on the receptor-binding domain

An indirect ELISA test based on the receptor-binding domain (RBD) of the virus was performed as a screening test (Raybiotech, Georgia, USA). The ELISA was adapted to each species by using a specific anti-species conjugate. Briefly, coated plates were covered with 100 μL of diluted sera (1/40) in PBS containing 0.05% Tween 20 (PBS-T) and incubated at 37° C for 30 min. The plates were then washed four times, 100 μL of the specific anti-species HRP-conjugated IgG (Jackson Immuno Research Laboratories, Cambridgeshire, UK) diluted 1/18,000 in PBS-T was added, and the solution was incubated at 37° C for 15 min. Four washes later, 100 μl of SureBlue Reserve TMB Microwell Peroxidase Substrate (TMB) (KPL, Gaithersburg, MD, USA) were added, and the plates were incubated in the dark, for 10 min. The reaction was stopped by adding 100 μl of 3M H2SO4 to each well. Absorbance at 450 nm was determined using an Anthos 2001 plate reader (Labtec, Salzburg, Austria). The endpoint cut-off was determined by the analysis of a receiver operating characteristic (ROC) curve based on positive divided by negative (P/N) values. Validation of this ELISA test is extensively described in (39).

### Virus neutralization test for detection of specific neutralizing antibodies against SARS-CoV-2

Virus neutralization test (VNT) was used to confirm the presence of neutralizing antibodies against SARS-CoV-2 in all the sera collected.

Briefly, the VNT was performed in duplicate in 96-well-plates by incubating 25 μL of two-fold serially diluted sera with 25 μL of 100 TCID50/ml of SARS-CoV-2. The virus-serum mixture was incubated at 37°C with 5% CO2. At 1-h post-incubation, 200 μL of Vero E6 cell suspension were added to the virus-serum mixtures, and the plates were incubated at 37°C with 5% CO2. The neutralization titers were determined at 3 days post-infection. The titer of a sample was recorded as the reciprocal of the highest serum dilution that provided at least 100% neutralization of the reference virus, as determined by the visualization of cytopathic effect (CPE). In addition, at the end of the period (3 days post-infection), cells were fixed with 6% paraformaldehyde and then stained with crystal violet to observe the cytopathic effect.

## Results

### SARS-CoV-2 infection prevalence

SARS-CoV-2 RNA was detected by RT-qPCR in seven cats and one dog by both “Diagnosis PCR” and “Omicron PCR.” This represents 10.13% of the total analyzed animals. All of the positive animals were sampled in Madrid and all their positive samples for RT-qPCR were negative for viral isolation (Table 1).

### Clinical signs

None of the animals that were part of this study presented any clinical signs at any time either during the quarantine time of their owners or afterwards.

### Antibody detection by employing the ELISA based on the RBD

Sera were collected from 15 animals (1 cat and 14 dogs), including Dog_8 and Cat_13 which were also positive for RT-qPCR (15 and 20 days after RT-qPCR positive result, respectively). However, none of the animals showed antibodies.

### Neutralizing antibodies detection by VNT

Among the 15 serum samples collected including both dogs and cat, none of them presented neutralizing antibodies.

### Whole-genome sequencing and phylogenetic analysis

**Figure 1:**
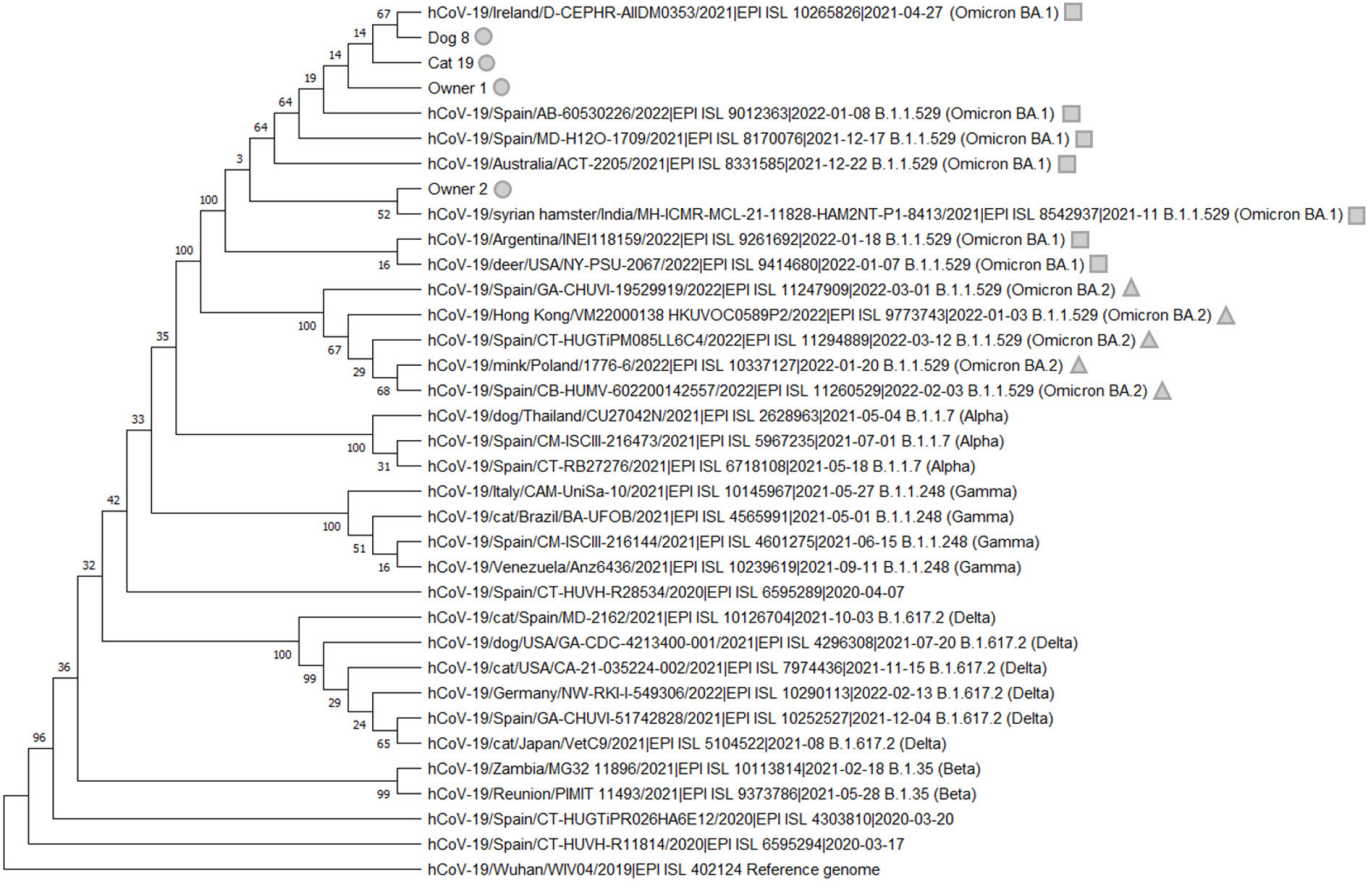
Phylogenetic analysis of SARS-CoV-2 of the whole-genome sequences from Dog_8, Cat_19, Owner_1, and Owner_2 (gray circle), which were clustered with the SARS-CoV-2 B.1.1.529 (Omicron) and more specifically with lineage BA.1 (gray square). The lineage BA.2 is indicated with a gray triangle. We appreciatively acknowledge the different laboratories and funders of GISAID for offering these SARS-CoV-2 sequences (Supplementary material 1).

The complete genome sequence of SARS-CoV-2 was obtained from the oropharyngeal swabs from both Dog_8 and Cat_19 (GenBank accession numbers: ON115270 and ON115269; GISAID accession ID: EPI_ISL_11580532 and EPI_ISL_11580576) as well as from the owner of Dog_8 (Owner_1; GenBank accession numbers: ON115271; GISAID accession ID: EPI_ISL_11580604) and the owner of Cat_26, 27 and 28 (Owner_2: GenBank accession number: ON115272; GISAID accession ID: EPI_ISL_11580636) since the remaining PCR-positive animals had too low viral RNA loads for effective sequencing (Figure 1).

After the construction of the phylogenetic tree, we visually verified that the Omicron lineage detected in this study in the dog and cat sequenced as well as the two owners correspond to BA.1 lineage, which it was the dominant in Spain by that date.

### Analysis of mutations in Omicron SARS-CoV-2

Analysis in the CoVsurver mutations app (GISAID) showed that the sequences presented several mutations having as a reference the hCoV-19/Wuhan/WIV04/2019 sequence. The mutations were 37 in the case of Dog_8 and Cat_19 (Tables 2, 3). No variabilities were observed at the nucleotide/amino acid level between the sequences from Dog_8 and its owner (Owner_1).

**Table 2:** List of mutations displayed in the different regions of the genome of SARS-CoV-2 in the sequence obtained in this study of Dog_8.

**Location in the genome**	**Mutations displayed**
NSP3 (ORF1a)	K38R, P985S, V1069I, S1265del, L1266I, A1892T
NSP4 (ORF1a)	T492I
NSP5	P132H
NSP6 (ORF1a)	L105del, S106del, G107del, I189V
NSP12 (ORF 1b)	P323L
NSP14 (ORF 1b)	I42V
Spike	A67V, H69del, V70del, T95I, G142D, V143del, Y144del, Y145del, N211del, L212I, ins214EPE,G339D, S371L, S373P, S375F,K417N, N440K, G446S, S477N, T478K, E484A, Q493R, G496S, Q498R, N501Y, Y505H,T547K, D614G, H655Y, N679K, P681H, N764K, D796Y, N856K, Q954H, N969K, L981F
E	T9I
M	D3G, Q19E, A63T
N	P13L, E31del, R32del, S33del, R203K, G204R

*NSP, Non-structural protein; E, envelope protein; M, Membrane protein; N, Nucleocapside protein*.

**Table 3:** List of mutations displayed in the different regions of the genome of SARS-CoV-2 in the sequence obtained in this study of Cat_19.

**Location in the genome**	**Mutations displayed**
NSP3 (ORF1a)	K38R, P985S, V1069I, S1265del, L1266I, A1892T
NSP4 (ORF1a)	T492I
NSP5	P132H
NSP6 (ORF1a)	L105del, S106del, G107del, I189V
NSP12 (ORF 1b)	P323L
NSP14 (ORF 1b)	I42V
Spike	A67V, H69del, V70del, T95I, G142D, V143del, Y144del, Y145del, N211del, L212I, ins214EP, G339D, S371L, S373P, S375F, K417N, N440K, G446S, S477N, T478K, E484A, Q493R, G496S, Q498R, N501Y, Y505H, T547K, D614G, H655Y, N679K, P681H, N764K, D796Y, N856K,Q954H, N969K, L981F
NS3	T14del, L15del
E	T9I
M	D3G, Q19E, A63T
NS7a	ins45EstopLN
N	P13L, E31del, R32del, S33del, R203K, G204R

*NSP, Non-structural protein; NS3, Non-structural protein 3; E, envelope protein; M, Membrane protein; NS7a, Accessory protein 7a; N, Nucleocapside protein*.

## Discussion

The SARS-CoV-2 B.1.529 (Omicron) variant, the last VOC detected, is nowadays highly disseminated around the world. Definitively in Spain, epidemiological data from the Omicron-associated wave has shown that the transmission rate of this variant is quite superior to other variants such as Beta or Delta. This fact has promoted the rapid spread of this variant, being dominant since November 2021 (10). One concern about this new variant is its potential transmission to other species, in which it could evolve and acquire new mutations that may be involved in higher virulence, among other fears. For this reason, it is necessary to evaluate its capability to infect susceptible species. In this sense, pets such as cats and dogs should be a major focus due to their close contact with humans.

In this study, we detected the Omicron SARS-CoV-2 variant in companion animals, demonstrating that pets are susceptible to the natural infection with this strain. However, the outcomes of this study revealed a relatively low number of positive animals based on RT-qPCR, given that the study involved an active sampling. In all the cases, owners assured high contact with their pets. In addition, the sampling was done at the best time for the detection of the disease (25) and only a10.13% of animals became infected, and no clinical signs were observed in any of them. These results contrast with previous reports in which the susceptibility of cats and dogs to other SARS-CoV-2 variants such as Alpha and Delta seems to be higher (31, 32, 40, 41). Furthermore, in the case of animals naturally infected with these other variants, clinical signs were described (32, 40, 42–44) and higher viral loads were detected (31, 32). In addition to this present study which assesses the natural infection of animals with this new variant, experimental infection studies have been carried out in cats which corroborate the absence of symptoms compared to other variants as well as lower viral loads (29). With respect to the owners, 70% of them were sampled and analyzed, and all of them were found to be positive for Omicron by “Omicron PCR” and/or sequencing. Taking into account the dates in which the sampling was performed, Omicron was the circulating variant in the different locations included in this study. The report issued by the Spanish Ministry of Health shows that from week 52 of 2021 (20^th^ December 2022), the Omicron variant was the most abundant variant in the human population based on all samples sequenced. Specifically, we know that the analyzed samples belong to the Omicron variant BA.1 lineage because of two things: the dates of appearance of this variant in Spain as well as the analysis of the mutations present in our sequences that coincide with the mutations of the BA.1 lineage (45). The Omicron variant also continues to be the most abundant variant circulating in the whole country until week 21 of 2022 (23^th^ May 2022), with updated data up to that date (46).

Another remarkable difference observed in animals infected with the Omicron variant is that viral isolation was not possible from any sample, due to the low viral load of all positive specimens. The fact that viral isolation was not possible could be due, in part, to the lower fusogenicity of this variant with respect to other variants (26) which may inhibit the virus entry into the cell. By contrast, viral isolation from cat and dogs samples has been possible in the case of the original virus isolate (39, 44) and other variants (31). Neither was it possible to detect neutralizing or non-neutralizing antibodies in any of the animals evaluated despite being exposed to positive people, nor the positive animals to RT-qPCR or the negative animals. This result contrasts with other SARS-CoV-2 seroprevalence studies in animals in which infection detected by RT-qPCR triggered an effective immune response based on neutralizing antibodies (25). This may be derived from the fact that virus replication may have been limited to a local level in the cases of this work. In consequence, it is possible that viral dissemination did not occur in the infected animals and the positive results were because of remnants of viral RNA (47). This could be explained by the fact that PCR-positive samples were only detected on 1 day of the 4 to 5 consecutive days of sampling.

All these results may be related to a higher affinity with the human cellular receptor which has been reported in the case of the Omicron variant compared to other variants (9, 10, 26). This could have led to the displacement of the binding between the animal cell and the virus, maybe due to specific variations in the ACE2 animal's receptor with respect to the human ACE2. This may be the reason for the variation of susceptibility in animals to this new variant compared to the previous ones. Further experimental research should be conducted to corroborate this hypothesis since experimental infection studies on cats and dogs with the Omicron variant are not reported so far.

However, these results contrast with those of an experimental study carried out in mink (30), in which high pathogenicity of the Omicron variant was observed, both at the level of symptoms and lesions. This higher susceptibility may be affected by the fact that mink-derived SARS-CoV-2 strains encode substitutions in areas of the genome crucial for ACE2 receptor binding that may enhance the binding of the spike protein to this receptor (48). It is, therefore, necessary to carry out studies on the pathogenicity of this variant in different animal species, as well as active surveillance to be able to early detection of new emerging variants.

Although so far there have been no publications on the presence of the Omicron variant in pets, it has been detected in wildlife, specifically in white-tailed deer (*Odocoileus virginianus*), which have been shown to be highly susceptible to the SARS-CoV-2 virus. Fortunately, despite their higher susceptibility, the risk of high contact with an infected human in this species is quite low, contrary to what is happening in the case of pets. These aspects highlight the importance of the investigation of these new variants both in urban and wild fauna (49).

From what we have observed in this study, it appears that the Omicron variant is less virulent to pets than the previous variants as well as the original isolate. Although 10.13% of the animals analyzed in this field study tested positive for RT-qPCR, low viral loads were detected and none of the infected animals showed any symptomatology according to their owners. This, together with our results previously obtained on other VOCs in animals (31, 32), has demonstrated the great variability of pathogenicity and response of each animal species to the different SARS-CoV-2 variants and the efficiency of our active surveillance system. This highlights the importance of conducting active surveillance both in pets living with COVID19 infected people and wildlife, in addition to genomic research to early detect infections with other variants or mutations associated with animal hosts. This also underlines the relevance of establishing a network of clinics and owners to be able to carry out active surveillance sampling.

## Data availability statement

The datasets presented in this study can be found in online repositories. The names of the repository/repositories and accession number(s) can be found in the article/Supplementary material.

## Ethics statement

The animal study was reviewed and approved by Complutense University of Madrid's Ethics Committee for Animal Experiments (Project License 14/2020). Written informed consent was obtained from the owners for the participation of their animals in this study.

## Author contributions

SB-A and JS-V designed the study. SB-A and LS-M performed the sampling, veterinary inspection, laboratory analysis, and wrote the initial manuscript. LD and JS-V acquired the funds. LD, MP-S, and JS-V reviewed the manuscript. All authors contributed to the article and approved the submitted version.

## Funding

This research was funded by the Institute of Health Carlos III (ISCIII) Project-Estudio del potencial impacto del COVID-19 en mascotas y linces (reference: COV20/01385) and the REACT ANTICIPA-UCM (reference PR38/21) funded by the Community of Madrid and the European Union through the ERDF (European Regional Development Fund) as part of the Union's response to the COVID-19 pandemic.

## Conflict of interest

The authors declare that the research was conducted in the absence of any commercial or financial relationships that could be construed as a potential conflict of interest.

## Publisher's note

All claims expressed in this article are solely those of the authors and do not necessarily represent those of their affiliated organizations, or those of the publisher, the editors and the reviewers. Any product that may be evaluated in this article, or claim that may be made by its manufacturer, is not guaranteed or endorsed by the publisher.
